# Epigenetic modifications of the *PHOX2A* and *CDH2* genes expression– new insights into the pathogenesis of multiple myeloma

**DOI:** 10.1186/s12885-025-15030-x

**Published:** 2025-10-27

**Authors:** Karolina Łuczkowska, Martyna Brzosko, Patrycja Stodolak, Piotr Kulig, Krzysztof Sommerfeld, Iga Stukan, Bartłomiej Baumert, Alina Jurewicz, Andrzej Bohatyrewicz, Edyta Paczkowska, Bogusław Machaliński

**Affiliations:** 1 Department of General Pathology, Pomeranian Medical University, Szczecin, Poland; 2 Department of Hematology and Transplantology, Pomeranian Medical University, Szczecin, Poland; 3 Department of Orthopedics, Traumatology and Oncology of the Musculoskeletal System, Pomeranian Medical University, Szczecin, Poland; 4 The Department of Specialized Nursing and Emergency Medical Care, Pomeranian Medical University, Szczecin, Poland; 5 Pharmaceutical Facility of Pomeranian Medical University, Szczecin, Poland

**Keywords:** Multiple myeloma, MGUS, Epigenetics, MiRNA, DNA methylation, *PHOX2A*, *CDH2*

## Abstract

**Introduction:**

Multiple myeloma (MM) is an incurable malignancy that arises from precursory conditions, specifically monoclonal gammopathy of unknown significance (MGUS) and smoldering multiple myeloma (SMM). The pathogenesis of MM remains largely elusive, particularly in the context of epigenetics.

**Purpose:**

The aim of this study was to uncover new bioinformatic insights related to the pathogenesis of MM in the context of epigenetic analysis using the NGS method.

**Patients and methods:**

A total of 60 patients with MM and MGUS were enrolled in the study. Myeloma CD138 + cells were isolated from the collected bone marrow using an immunomagnetic method and used to analyze the DNA methylation profile using the MethylationEPICv2.0 BeadChip Kit. Peripheral blood plasma was used to analyze the expression profile of circulating miRNAs using the miRNAseq method. Additionally, global epigenetic assessment allowed for the selection of several target genes and assessment of their expression using the qRT-PCR method.

**Results:**

Our in-depth analysis allowed us to focus on two genes, *PHOX2A* and *CDH2*, which play significant roles in carcinogenesis, and their increased expression is associated with poor prognosis in oncological patients. We observed a decrease in the methylation level associated with these genes in patients with MM compared with those with MGUS, whereas the mRNA expression level was increased. Moreover, among patients with MGUS compared with MM, patients with MGUS presented upregulation of specific miRNAs, namely, miR-208b-3p, and miR-320c, which act as inhibitors of the aforementioned genes.

**Conclusions:**

Ultimately, identifying genes implicated in the progression of MM may pave the way for the refinement of current treatment protocols or the development of novel therapeutic strategies centred on epigenetic modifications or gene therapies. Additionally, the expression profile of circulating miRNAs may prove useful in selecting molecules that will constitute a good biomarker of disease progression from the preclinical to the fully symptomatic stage.

## Introduction

Multiple myeloma (MM) is a type of monoclonal plasma cell neoplasia. MM is characterized by bone marrow infiltration or solitary tumour formation by terminally differentiated plasma cells, the toxicity of which results from direct harmful effects and excessive excretion of nonfunctional monoclonal immunoglobulins and cytokines [1, 2]. Current treatment regimens are based on the use of several drugs, often in combination with haematopoietic stem cell transplantation. Contemporarily used drugs belong mainly to the following groups: a) immunological; b) proteasome inhibitors; c) monoclonal antibodies; d) bispecific antibodies; and e) CAR-T-cell therapy [3]. Myelomagenesis begins with potentially premalignant and asymptomatic monoclonal gammopathy of undetermined significance (MGUS), which progresses to smoldering myeloma and ultimately symptomatic MM. Malignant transformation is a complex process that requires both genetic and environmental transformation [4]. To diagnose MGUS, the infiltration of plasma cells in the bone marrow cannot exceed 10%, and there are no signs of organ damage, i.e., the CRAB criteria are not met (this acronym stands for hypercalcemia (C), renal failure (R), anaemia (A), and bone lesions (B) [2] (Table 2)). However, in recent years, a group of MGUS patients with certain clinical manifestations have been characterized and given the name monoclonal gammopathy of clinical significance (MGCS). Patients with MGCS present conditions similar to those of patients with MGUS but are also characterized by damage to the kidneys, peripheral nerves, or skin. In cases where MGCS is accompanied by renal or neurological impairment, the condition should be more precisely classified as monoclonal gammopathy of renal significance (MGRS) and monoclonal gammopathy of neurological significance (MGNS), respectively [5, 6].

The risk of developing the symptomatic stage of myeloma depends on the concentration of monoclonal protein, the number of plasma cells in the bone marrow, the ratio of free immunoglobulin light chains, and the presence of immunoparesis [2]. Emerging evidence highlights the crucial importance of epigenetic alternations in the development of malignancy [4, 7]. Methylation changes, such as gradual loss of non-CpG methylation and progression-related changes in CpG methylation, occur during normal B-cell maturation [8]. Barwick et al. reported that while normal B cells and normal plasma cells had median global CpG methylation levels of 71% and 89%, respectively, a dramatic hypomethylation of 41% was detected in myeloma samples. DNA methylation remains mostly unchanged in the bodies of genes that are highly expressed [9]. Similar changes are noticeable during the transition from MGUS to MM [10]. Epigenetic deregulation in multiple myeloma is observed in the form of mutations or aberrant expression of *DMETs* (*DNMT1/3A/3B*) and *TETs* (*TET1/2/3*) [11], histone modifiers (HDAC class I [12], HDAC6 [13], MMSET [14], EZH2 [15], PRMT5 [14], KDM3 [16]), and noncoding RNAs [14–17]. DNA methylation influences gene expression patterns and overall genome stability [18]. Hypermethylation can be found not only in tumour suppressor genes such as *TP53, CDKN2B, CDKN2A, and SAPK1* and in members of the WNT and JAK/STAT signalling pathways [7, 19] but also in B-cell-specific genes and transcription factors [18–21]. Hypomethylation remains within repetitive sequences (e.g., LINE-1, Alu and SAT-A) and transposable elements [10, 18, 22, 23]. Certain methylation patterns may be specific to cytogenetic variants, e.g., the highest frequency of hypermethylated genes can be found in the t(4;14) translocation subgroup, which corresponds to 15–20% of the MM population [10, 18].

A better understanding of the biology of the disease has led to significant improvements in treatment options, such as the introduction of proteasome inhibitors, immunomodulatory drugs, and monoclonal/bispecific antibodies. There have also been attempts to use chimeric antigen receptor T-cell therapy (CAR-T) [24]. However, all treatments are limited by the plasticity of cancer cells and the development of resistance [18, 25]. Understanding epigenetic changes in MM development could lead to the development of new targets for MM treatment [7] and prognostic markers for MGUS patients. Although, as mentioned above, it is relatively well established that epigenetics plays a role in cancer development and progression, the exact epigenetic mechanisms driving MGUS to MM remain to be determined. Therefore, the aim of this study is to shed more light upon the epigenetic aspects of MGUS to MM progression using advanced bioinformatic analytic tools.

## Materials and methods

### Subjects and initial management

The study focused on patients diagnosed with MM and MGUS who were enrolled in the Department of Haematology and Bone Marrow Transplantation at Pomeranian Medical University in Szczecin, Poland. To ensure the reliability of the findings, individuals with a history of other malignancies were excluded from the research. Furthermore, all patients with MGUS were asymptomatic and did not present clinically significant differences (MGCSs). The clinical characteristics of the patients are detailed in Table 1 and Table 2.

**Table 1:** Characteristics of patients with MM and MGUS

Clinical characteristics	MM	MGUS
Sex (F/M)	10/20	21/9
Age (mean, ± SD)	64.26 ± 11.52	68.30 ± 8.67
Hypertension (No/Yes)	17/13	14/16
Diabetes mellitus (No/Yes)	27/3	23/7
Ischemic heart disease (No/Yes)	28/2	27/3
Hypercholesterolemia (No/Yes)	25/5	24/6
Liver diseases (No/Yes)	30/0	29/1
Autoimmune diseases (No/Yes)	28/2	17/13
Respiratory diseases (No/Yes)	26/4	28/2

**Table 2:** Prevalence of CRAB and SliM criteria in patients with MM

	Number of patients (Yes/No/no data)	Percentages (Yes/No/no data)
C	6/24/0	20%/80%/0%
R	6/24/0	20%/80%/0%
A	15/15/0	50%/50%/0%
B	27/3/0	90%/10%/0%
S	6/24/0	20%/80%/0%
Li	10/20/0	33%/66%/0%
M	17/2/11	56.7%/6.7%/36.6%

(CRAB acronym: hypercalcemia (C), renal failure (R), anemia (A), and bone lesions (B); SLiM acronym: ≥ 60% clonal bone marrow plasma cells (S); ≥ 100 serum light chain I/U ratio (Li); ≥ 1 focal bone lesion of ≥ 5 mm in size (MRI lesions) (M). The numbers of patients are presented in the order Yes/No/no data and as percentages

Peripheral blood and bone marrow samples were collected in EDTA tubes and centrifuged immediately with a double spin to remove cells and debris. RNA was isolated immediately from the 700 µl plasma volume using the same extraction kit for all samples. The isolated miRNA was stored at –80 °C. Visibly hemolyzed samples were excluded. The procedures for isolating CD138^+^ cells from the bone marrow were undertaken as quickly as possible, and the isolation of genetic material was performed immediately after CD138^+^ cells were obtained. All enrolled patients in the MM group were untreated at the time of bone marrow and plasma collection. The study was approved by the local bioethics committee (KB-0012/48/19), and before enrolment in the study, all patients signed informed consent in accordance with the Declaration of Helsinki.

**Fig. 1:**
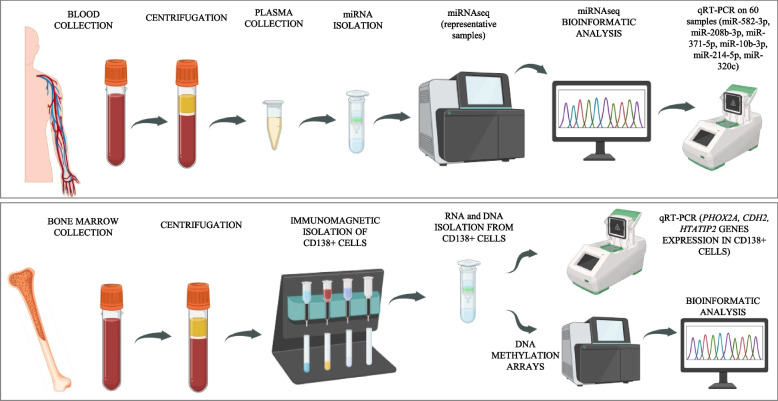
Experiment scheme created with biorender

Owing to the complexity and significant costs of the selected procedures, the following division of analyses was used. For global DNA methylation and circulating miRNA profiling, six samples were selected from each patient group (MM and MGUS). The selected samples were representative in terms of sex, age, and presence of comorbidities. In the MM group, the representative samples included 2 females and 4 males aged 50–75 years, covering patients both with and without comorbidities: hypertension (3/3), diabetes mellitus (1/5), and autoimmune diseases (1/5). The selected MM samples also reflected CRAB/SLiM criteria, including A (3/3), Li (2/4), ensuring that the subset captured the diversity of clinical manifestations within the cohort. In the MGUS group, the representative samples included 4 females and 2 males aged 60–74 years, covering patients both with and without comorbidities: hypertension (3/3), diabetes mellitus (1/5), hypercholesterolemia (2/4), and autoimmune diseases (2/4). This selection ensured that the chosen samples reflected the distribution of sex, age, and major comorbidities in the overall cohort, minimizing selection bias and enhancing the reliability of the omics analyses. More homogeneous features (e.g., hypercalcemia, bone lesions) were not prioritized, as all or nearly all patients share these characteristics, providing Little discriminatory value After bioinformatic analysis, the 6 miRNAs with the greatest variability were selected, and expression analysis was subsequently performed via qRT‒PCR on 30 plasma samples from each group. Additionally, the expression of three genes that presented the greatest variability in DNA methylation was assessed to verify the actual impact of the observed epigenetic changes on transcription (Fig. 1).

### Immunomagnetic separation

A bone marrow (BM) sample (∼12 mL) was collected in EDTA tubes from the posterior iliac crest. The BM was subsequently lysed via BD Pharm Lys Lysing Buffer (Becton Dickinson, Franklin Lakes, NJ, USA) according to the manufacturer's protocol. Bone marrow cells were subjected to immunomagnetic isolation to obtain the CD138^+^ cell population. For this purpose, a CD138 MicroBeads kit, human (Miltenyi Biotec, Auburn, AL, USA), was used, and the manufacturer's protocol was followed.

### DNA extraction and bisulfate conversion

DNA was isolated from CD138^+^ cells via the PureLink Genomic DNA Mini Kit (Thermo Fisher, Waltham, MA, USA), and RNA was isolated via the PARIS Kit (Thermo Fisher Scientific, MA, USA). In both cases, isolation was carried out according to the manufacturer's protocol. The isolated DNA and RNA samples were assessed via a TapeStation 4510 (Agilent Technologies, Santa Clara, CA, USA). All the samples met the quality criteria (DINs ≥ 9 and RINs ≥ 9). Bisulfate conversion was carried out via the EZ DNA Methylation-Gold Kit (Zymo Research, Irvine, CA, USA) according to the manufacturer's protocol. Five hundred nanograms of DNA from each sample was used for conversion.

### miRNA extraction

Peripheral blood (~ 7.5 mL) was collected into EDTA tubes and then centrifuged for 10 min at 2000 rpm to obtain plasma free of blood morphotic elements. Then, the plasma was transferred to a new tube and centrifuged again under the same conditions. The aim of double centrifugation was to completely remove nucleated blood cells from the plasma, which are also a source of miRNA and could distort the test results. miRNA circulating in plasma was isolated from 700 µl of plasma via the NucleoSpin miRNA Plasma Kit, Mini Kit for Circulating miRNA (Macherey–Nagel, Düren, Germany). miRNA isolation was performed according to the manufacturer's recommendations. The isolated material was eluted from the column with water and suspended in a final volume of 15 µl. The miRNA concentration was measured via a Qubit 4 Fluorometer (Thermo Fisher Scientific, Waltham, MA, USA) and a Qubit™ microRNA Assay Kit (Thermo Fisher Scientific, Waltham, MA, USA).

### Methylation arrays

The DNA methylation profile was analysed via the Infinium MethylationEPICv2.0 BeadChip Kit, human (Illumina, San Diego, CA, USA). Methylation arrays were prepared in strict accordance with the manufacturer's instructions. The arrays were scanned via a NextSeq550 instrument (Illumina, San Diego, CA, USA).

### miRNA-seq

TruSeq Small RNA Library Preparation Kits (Illumina, San Diego, CA, USA) and NextSeq 500/550 v2.5 Kits (mid-output, 150 cycles) (Illumina, San Diego, CA) were used to analyse the global expression of circulating miRNAs in plasma. The procedure was carried out in accordance with the manufacturer's protocol. After library preparation, each sample was gel purified. Bands of ~ 147 bp were cut from the gel, which contained purified miRNAs along with adapter sequences. The elution of miRNA from the gel was performed via dedicated gel breaker tubes and ultrapure water.

### qRT‒PCR

The expression analysis of selected genes and miRNAs was performed via qRT‒PCR. The genes and miRNAs were selected based on the results obtained from the methylation array and miRNA-seq. The main criterion was the high variability of methylation/expression levels between the MM and MGUS groups and the association with possible disease progression. qPCR was performed via a ready-made SYBR Green PCR Master Mix kit (Bio-Rad, Hercules, CA, USA). The reaction was carried out on a Bio-Rad CFX96 Real-Time PCR Detection System (Bio-Rad Inc., Hercules, CA, USA). Expression analyses of all the samples were performed in duplicate (*n* = 2). For normalization of gene expression, *GAPDH* was selected as the reference gene, and for normalization of miRNA expression, *miR-93* was used as the endogenous control. The selection of these normalizers was based on previously published studies in MM and MGUS, where both *GAPDH* and *miR-93* were reported as commonly applied and stable reference molecules under similar experimental conditions [26–31].

#### miRNA expression

The primers were designed via the miRPrimer program, and their sequences are presented in Table 3. Reverse transcription was performed via a Mir-X™ miRNA qRT‒PCR kit (Takara Bio, Kusatsu, Shiga, Japan). miRNAs expression was performed in technical duplicates for each patient sample. Biological replication was ensured by the inclusion of 30 independent patient samples with MM and 30 independent patient samples with MGUS, each of which constituted a separate biological replicate.

**Table 3:** Primer sequence used in the qRT-PCR reaction

Gene/miRNA	Primer Sequences
*PHOX2A*	F 5′- GGCAGTGCCCTACAAGTTCT-3′ R 5′-GGAACCAGACCTGCACGC-3′
*CDH2*	F 5′-AGGCTTCTGGTGAAATCGCA-3′ R 5′-GGAGGGATGACCCAGTCTCT-3′
*HTATIP2*	F 5′-GGGAAGGTGGGATGCTCTGA-3′ R 5′-CCATTCACCTGGGCGAGATT-3′
miR-582-3p	F 5′-CAGTAACTGGTTGAACAACTGA-3
miR-208b-3p	F 5′-GCAGATAAGACGAACAAAAGGT-3′
miR-371b-5p	F 5′-TCAAAAGATGGCGGCAC-3′
miR-10b-3p	F 5′-GCAGACAGATTCGATTCTAGG-3′
miR-214-5p	F 5′-TGCCTGTCTACACTTGCT-3′
miR-320c	F 5′-CAGAAAAGCTGGGTTGAGAG-3′

#### Gene expression

The primers were designed via the BLAST program, and their sequences are presented in Table 3. Reverse transcription was performed via a First Strand cDNA Synthesis Kit (Thermo Fisher Scientific, Waltham, MA, USA).

### Bioinformatics analysis

Bioinformatics analyses were performed in the R programming environment using appropriate Bioconductor libraries. Raw data from files (.idat) generated after array scanning were analysed via the "ChAMP" pipeline. Batch effects and other unwanted changes were removed via the "sva" bioconductor library. The final linear models were generated from the “limma” package to calculate the corrected p value and beta value (fluorescence intensity ranged from 0 (unmethylated) to 1 (fully methylated)). For each CpG probe, a delta beta value corresponding to differential methylation in the compared groups was also calculated. Methylation scores with delta beta values > 0.2 were considered significant. The Benjamini–Hochberg test (FDR) was used to correct the p value. The results were visualized via the following R libraries: "ggplot2", "ggprism", "ComplexHeatmap" and "ggpubr". Differential expression of miRNAs was analyzed using the DESeq2 package in R, applying standard procedures for normalization and variance modeling of count data. miRNAs with adjusted *p*-value < 0.05 (Benjamini–Hochberg FDR correction) and absolute fold change ≥ 2 were considered significantly deregulated. Data visualization was performed using ggplot2, while miRNA–gene interaction networks were presented as interactive graphs using the networkD3 package. DAVID Bioinformatics Resources (Database for Annotation, Visualization, and Integrated Discovery) at http://david.abcc.ncifcrf.gov were used for functional annotation and enrichment analysis. The functional annotation charts generated by DAVID with overrepresented gene annotations are shown as bubble plots from the BACA BioConductor package (https://cran.r-project.org/web/packages/BACA/BACA.pdf) [32–34]. The criteria used to generate the charts were *p* < 0.05, method = Benjamini, beta value = 0.3, and minimum number of genes per group = 5. Genes whose methylation level increased in relation to the compared group are marked in green, and Genes whose methylation level decreased are marked in red. Mean TPM values across samples ranged from 608 to 2466, while the median was 0 in all samples, which is typical for miRNA-seq data where many miRNAs are expressed at very low levels. The number of expressed miRNAs (TPM > 0) ranged from 503 to 896, and total read counts per sample ranged from 451 thousand to 6 million. Analysis of TPM and counts distributions revealed no outlier samples, and mean counts were consistent across samples. These results confirm the high quality of the miRNA-seq data and their suitability for downstream differential expression analysis.

### Statistical methods

Gene expression analysis was performed via CFX Maestro Software (Bio-Rad, Hercules, CA, USA) via the 2ΔCt method. Student's t test was used to analyse statistical significance (*p* value < 0.05). Statistical power was estimated for a two-sample t-test (two-tailed; α = 0.05; *n* = 30 per group). Under these parameters, 80% power is achieved for an effect size of Cohen’s d = 0.736. With Bonferroni correction for six tests (α = 0.0083), the required effect size increases to d = 0.93. Observed standardized effects for the studied miRNAs ranged from d ≈ 0.45 to 0.71, corresponding to achieved power of ~ 0.41–0.77 (without multiple testing correction).

## Results

### Epigenetic profiling by DNA methylation array in MM and MGUS

Global methylation analysis allowed the assessment of 847 462 sites in the Genome. Bioinformatics analysis revealed a decrease in global methylation levels in MM patients. A total of 250 genes with reduced methylation were observed in MM compared with MGUS, which confirms a significant difference between these two stages of the disease. Further bioinformatic analysis allowed the selection of several genes that had not previously been attributed to the pathogenesis of MM. The *HTATIP2* tumour suppressor gene is characterized by an increased level of methylation in MM patients compared with patients with MGUS (Table 4), which (as we showed later in the study) resulted in a decrease in its expression (Table 5). Genes whose methylation levels are lower in patients with MM than in those with MGUS, such as *PHOX2A, CDH2, ADAMTS16, BTBD3,* and *IRS2*, are responsible for cancer progression, and their increased expression is correlated with poor prognosis, reduced survival time, and increased frequency of metastases. In this study, we established a relationship between a decrease in methylation and an increase in gene expression and vice versa. The decreased level of methylation of the *PHOX2A* and *CDH2* genes (Table 4) in patients with MM resulted in an increase in their expression (Table 5), whereas the increased level of methylation of these genes (Table 4) in the MGUS group resulted in the inhibition of their expression (Table 5). Hypermethylation of the *PHOX2A* and *CDH2* genes in MGUS patients was identified in the CpG island region of the promoter, precisely at the transcription start sites TSS1500 and TS200.

**Table 4:** Mean beta-values of 30 genes in CD138^+^ cells with the highest variability

Gene	MGUS	MM	MGUS vs. MM	*p-value*
*PHOX2A*	0.501	0.101	0.400	0.002
*C5orf38*	0.533	0.134	0.399	0.002
*IRS2*	0.547	0.166	0.380	0.001
*GSN-AS1*	0.621	0.253	0.368	0.002
*CDH2*	0.494	0.128	0.366	0.006
*ADAMTS16*	0.504	0.138	0.366	0.004
*AHRR*	0.630	0.264	0.365	0.012
*BTBD3*	0.444	0.081	0.363	0.004
*C5orf38*	0.453	0.093	0.360	0.005
*TMTC1*	0.564	0.205	0.359	0.005
*FOXQ1*	0.487	0.133	0.354	0.009
*TBX20*	0.498	0.145	0.353	0.019
*BTBD17*	0.446	0.093	0.353	0.016
*SNTG2*	0.402	0.050	0.352	0.012
*UNCX*	0.428	0.077	0.352	0.016
*RPL17*	0.342	0.702	−0.360	0.016
*HTATIP2*	0.252	0.571	−0.319	0.012
*ZNF784*	0.552	0.865	−0.313	0.013
*GNA12*	0.377	0.674	−0.297	0.007
*KLHL35*	0.404	0.692	−0.289	0.034
*PSD2*	0.409	0.695	−0.286	0.016
*SPRING1*	0.278	0.556	−0.278	0.007
*DBNL*	0.437	0.712	−0.276	0.026
*COX18*	0.454	0.728	−0.274	0.009
*FDFT1*	0.454	0.721	−0.268	0.029
*ZNF329*	0.448	0.715	−0.267	0.036
*UNC13B*	0.318	0.585	−0.267	0.012
*DNAL1*	0.484	0.749	−0.266	0.025
*DNAH6*	0.174	0.439	−0.264	0.025
*H1-0*	0.252	0.516	−0.264	0.032

Negative values indicate decreased methylation levels in MGUS vs. MM patients. A beta-value of 0 means no methylation, a value of 1 means full methylation (*p*-values < 0.05; adj *p* < 0.99 for all genes)

**Table 5:** Real-time quantitation of selected genes *PHOX2A, CDH2, HTATIP2* in CD138^+^ cells (*p* < 0.05)

	*HTATIP2* (Mean ± SD)	*PHOX2A* (Mean ± SD)	*CDH2* (Mean ± SD)
MM	**0.57** ± 0.12	**7.34** ± 3.66	**53.92** ± 26.90
MGUS	**0.25** ± 0.16	**0.51** ± 0.07	**0.69** ± 0.01

*SD* Standard Deviation, bold indicates statistical significance both comparing study groups to controls and MGUS to MM

### Pathway and gene ontology analysis of differentially methylated genes

**Fig. 2:**
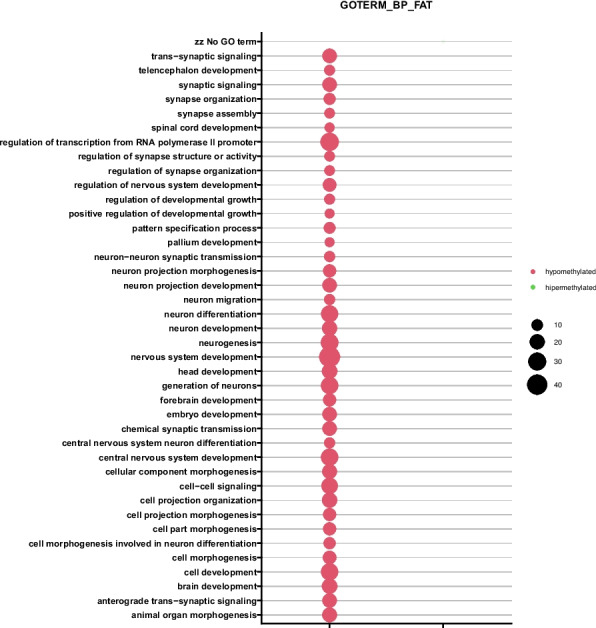
The bubble plot with altered methylation levels assigned according to Gene Ontology (GO) classification in CD138^+^ myeloma cells (MM vs. MGUS);* p* < 0.05

One of the most interesting changes generated by DAVID analysis was the regulation of transcription from the RNA polymerase II promoter. Genes regulating this process were hypomethylated in the MM group compared with the MGUS group (Fig. 2). Therefore, a decrease in the methylation level of genes regulating this process may have a significant impact on the progression of MM.

### miRNA expression profiles associated with disease progression

**Fig. 3:**
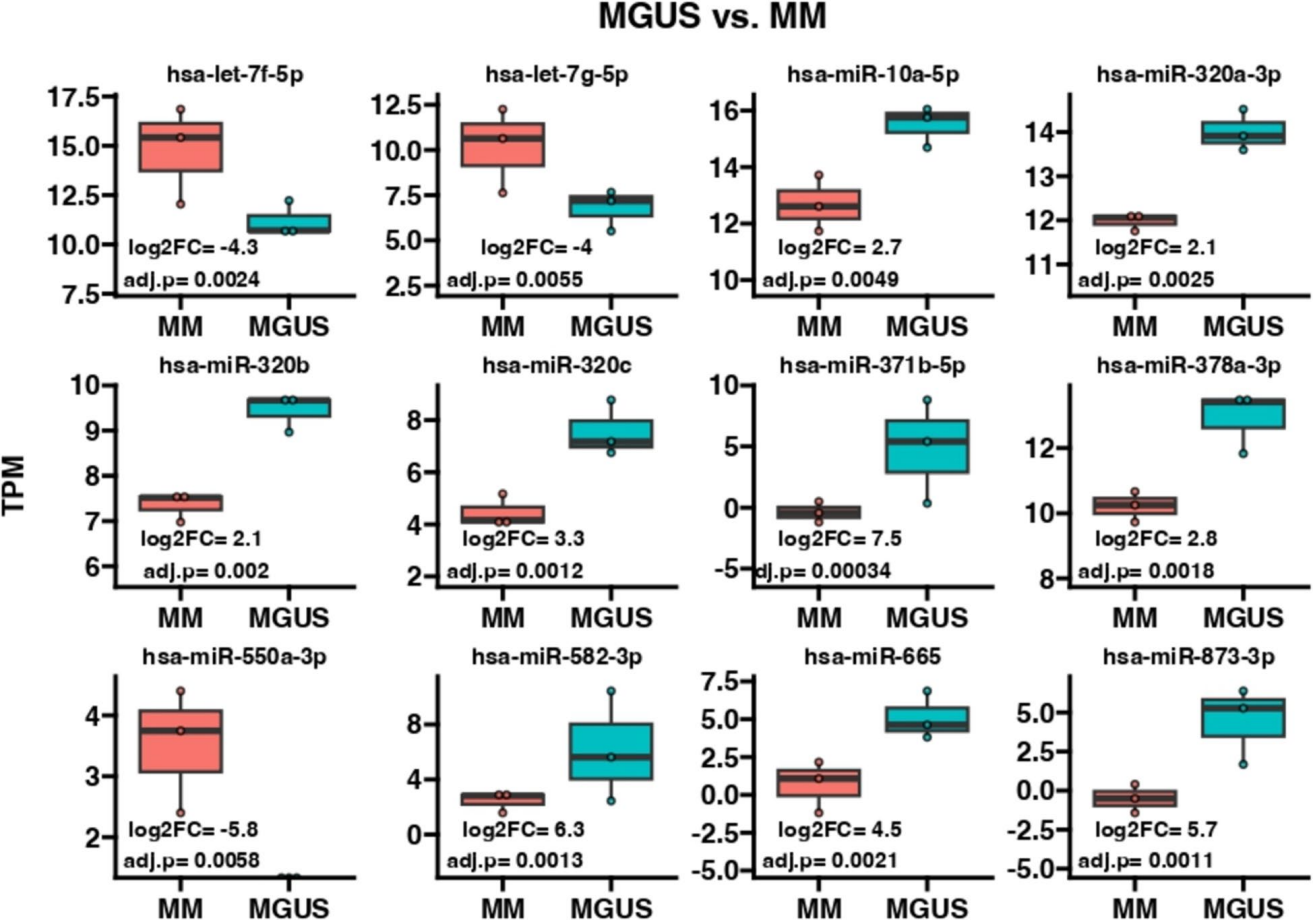
Expression of selected miRNAs circulating in the plasma of patients with MM and MGUS. The chart shows the variability value (log2FC) of comparable groups and the padj value

Bioinformatics analysis revealed 2656 variations in the expression levels of miRNAs circulating in plasma. A total of 40 miRNAs were compared between MGUS and MM (fold change > 2, padj < 0.1), among which 27 showed statistically significant differences with padj < 0.05. Figure 3 presents graphs of the expression levels of 12 selected miRNAs circulating in plasma in individual groups of patients recruited for the study. miRNAs were selected based on the largest statistically significant difference in expression in the compared groups (statistical significance is indicated in each graph). The log2FC value marked on the graph refers to the fold changes in the compared groups. Negative values indicate a decrease in expression, whereas positive values indicate an increase in expression. Important observations in patients with MGUS vs. MM include increases in the expression of miRNAs that are involved in cancer progression: hsa-miR-371b-5p; hsa-miR-10b-3p; hsa-miR-1291; hsa-miR-582-3p; hsa-miR-208b-3p; hsa-miR-873-3p; hsa-miR-665; hsa-miR-214-5p; hsa-miR-320c; hsa-miR-378a-3p;; and miR-550a-3p. miR-550a-3-5p is classified as a tumour suppressor; therefore, its expression is inhibited with the development of individual cancer stages. In addition, using the miRDB database, target genes for the above preselected miRNAs with the highest variability were obtained. These data were subsequently compared with the results from the methylation array, and three miRNAs were identified: miR320c, miR208b-3p, and miR582-5p, which regulate the expression of the *PHOX2A* and *CDH2* genes. The expression of these miRNAs is significantly greater in patients with MGUS, which results in the inhibition of the *PHOX2A* and *CDH2* genes (Table 4). With the development of the disease, the concentration of miRNAs (miR320c, miR208b-3p, and miR582-5p) decreases, which results in increased expression of the pro-oncogenic genes *PHOX2A* and *CDH2*. A similar relationship was observed in the case of the methylation of these genes; with the development of the disease, the methylation of these genes decreases which results in an increase in their expression. The analysis performed allowed for the selection of new relationships at the epigenetic level and their verification at the transcriptional level.

### Gene expression in relation to miRNA and DNA methylation changes

We selected genes (*PHOX2A, CDH2, and HTATIP2*) whose methylation levels significantly changed to validate their expression via qRT‒PCR in CD138^+^ myeloma cells (Table 5). The selected genes confirmed the actual impact of the methylation level on gene expression. A comparison of the methylation levels shown in Table 4 revealed that an increase in gene methylation resulted in a decrease in gene expression.

#### Validation of the expression of selected miRNAs

The expression (Table 6) of selected miRNAs (miR-582-3p, miR-208b-3p, miR-371b-5p, miR-10b-3p, miR-214-5p, and miR-320c) in the plasma of patients with MM and MGUS was assessed via qRT‒PCR. The obtained results confirm the relationships between the studied groups, which are provided by the miRNAseq analysis. miRNAs were selected based on miRNA-eq data. The selection criteria were both high expression variability and involvement in carcinogenesis and tumour progression.

**Table 6:** The relative expression level of selected miRNAs was measured by qRT-PCR in the plasma of patients with MM and MGUS

	MM (Mean ± SD)	MGUS (Mean ± SD)
miR-582-3p*	1.73 ± 3.28	3.32 ± 3.69
miR-208b-3p*	18.51 ± 51.11	467.30 ± 1397.77
miR-371b-5p*	56.68 ± 121.54	336.43 ± 594.74
miR-10b-3p*	4.84 ± 11.40	11.90 ± 8.16
miR-214-5p*	20.90 ± 39.40	74.16 ± 156.57
miR-320c*	2334.71 ± 1301.51	3241.27 ± 2380.50

*p < 0.05

#### Evaluation of predictive value of selected miRNAs using ROC curves

**Fig. 4:**
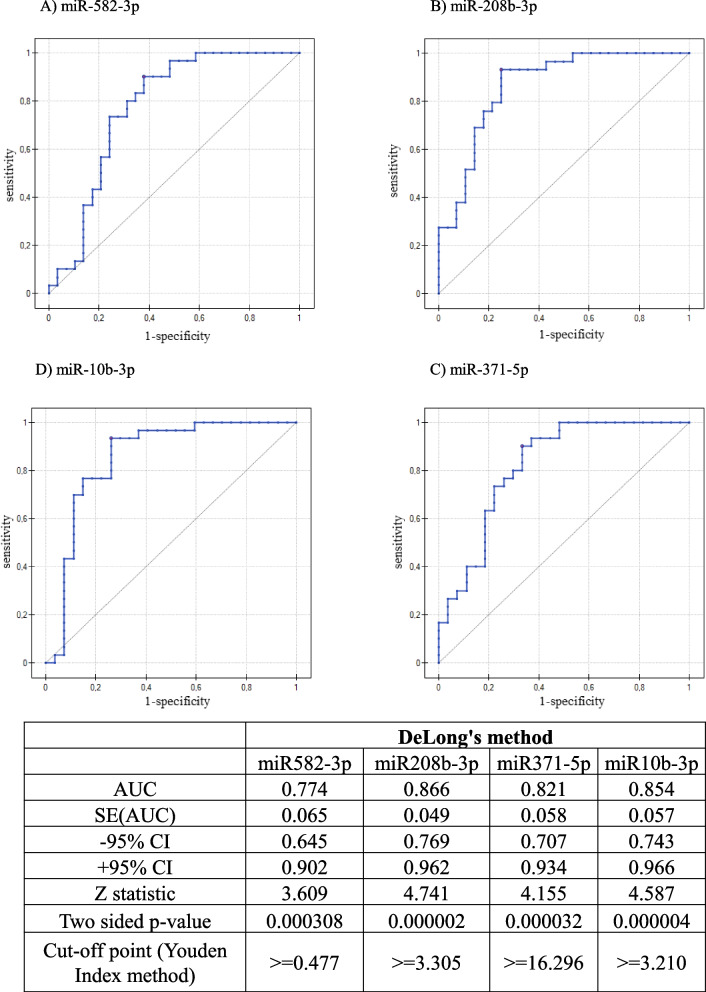
ROC curves

To select a potential biomarker for the development of the preclinical stage into full-symptomatic multiple myeloma, receiver operating characteristic (ROC) analysis was performed, which allows for the determination of the sensitivity and specificity of the tested factor. For this purpose, miRNA expression in the plasma of patients with MGUS and MM obtained via qRT‒PCR was analysed. The cut-off point was determined via the Youden index. Analyses and ROC curve analysis were performed via PQStat software. The analysis was performed on all the miRNAs assessed via qRT‒PCR; however, Fig. 4 shows only those miRNAs whose AUC was above 0.7. The remaining miRNAs had AUCs ranging from 0.58 to 0.69. The criteria defining the reliability of the biomarker were developed according to the available Literature, and it was assumed that an AUC of 0.5 indicates no discrimination and that a value of 1.0 indicates perfect discrimination in terms of sensitivity and specificity. It is most often assumed that a well-chosen model, useful diagnostically, takes values > 0.7. Therefore, AUC values of 0.9–1.0 were considered excellent biomarkers; 0.8–0.9, good; 0.7–0.8, fair; 0.6–0.7, poor; and 0.5–0.6, failure [35, 36]. The best potential biomarker was miR208b-3p (AUC = 0.866). The obtained AUC value indicates good diagnostic value. A promising approach for the analysis of miRNA biomarkers is the integration of blockchain, machine learning, and IoT, as described by Ghorbian et al. [37, 38], which could, in the future, complement traditional statistical methods such as ROC analysis, enhancing the prediction of disease progression and enabling more effective patient monitoring in multiple myeloma.

## Discussion

MM is the second most common haematological malignancy [39] and has a multifactorial pathogenesis that remains to be elucidated. Both conditions, i.e., MGUS and SMM, are considered premalignant predecessors of MM, with a risk of progression to symptomatic MM of 1% and 10% per year, respectively [40]. Although several mechanisms have been shown to be involved in myelomagenesis, the exact pathophysiology of the progression from MGUS to fully symptomatic MM has not yet been determined. For example, several oncogenes, such as the CYCLIN D gene, participate in this transition [41]. In addition, an increase in pathological angiogenesis has been shown to be an important factor in the transition from MGUS to MM [42]. In addition to well-established mutations and polymorphisms, epigenetic mechanisms are postulated to play a role. In this study, we investigated the methylation and miRNA profiles of both MGUS and MM patients. We aimed to shed more light on the epigenetic alterations that may participate in the complex pathogenesis of MM, with particular emphasis on the transformation of MGUS into MM. We showed that the methylation profile of MGUS patients differs from that of MM patients, which corresponds to generally available data in this regard [20]. MM cells were globally hypomethylated in comparison with MGUS plasma cells. Collectively, our results indicate that cells obtained from MGUS and MM patients exhibit different methylation profiles; therefore, one may hypothesize that the epigenetic footprint enables us to distinguish between the abovementioned states. In summary, global methylation levels tend to decrease as plasma cells transform into MM cells. Similar observations have been reported by other researchers. For example, Agirre et al. investigated the methylome of MM cells. First, they reported that the methylation pattern is different in normal plasma cells, MGUS and MM. Moreover, their results revealed that progression from MGUS to MM appears to be associated with a decrease in methylation alongside the absence of additional hypermethylation events [43]. Salhia and colleagues conducted an interesting study in which they investigated the role of methylation patterns in the genesis of myeloma. These authors reported that the methylation levels of MGUS and SMM did not differ (p = 0.47), but both conditions differed from those of MM (p < 0.0001). Notably, normal plasma cells had the highest median methylation level, which was significantly different from that of both premalignant and malignant successors (*p* < 0.0001). The study concluded that loss of methylation is a vital and early event in myelomagenesis, announcing the initiation and progression of MM [44]. Stanganelli and team conducted an interesting study that did not focus on the global methylation pattern but aimed to investigate the promoter methylation of various suppressor genes, such as *p16INK4A, p15 INK4B, ARF, p27KIP1, TP73, RASSF1A,* and *SOCS-1,* in patients with MGUS and MM. They reported that the methylation of *TP73, ARF, p15INK4B,* and *p16INK4A* is an early event in the pathogenesis and development of gammopathies. In parallel, *SOCS-1* methylation can be considered an important step in clonal evolution from MGUS to MM [45]. These findings suggest and are consistent with our results that the global and gradual loss of methylation is an epigenetic footprint that distinguishes normal plasma cells from plasmacytes obtained from patients with premalignant states, i.e., MGUS and SMM. MM is associated with the lowest methylation level. Moreover, the decrease in methylation may play a role in the clonal transformation of normal plasma cells into their malignant counterparts. In addition to genome-wide methylation analysis, by performing thorough bioinformatics analysis, we conducted further analysis and identified genes with significantly different methylation levels between MGUS and MM. We identified several hypermethylated genes in MM compared with MGUS. The role of these genes in myelomagenesis should be further investigated and is yet to be determined. For example, *RPL17* was shown to promote colorectal cancer [46]. We also detected hypermethylation of the Gene encoding zinc finger protein 784 (*ZNF784*). Its role in cancer biology is unknown; however, other Genes encoding zinc finger proteins have been demonstrated to participate in cancer progression and metastasis 50 [47]. In addition, we have shown that there are differences in the methylation levels of different genes. Compared with those in MGUS, the genes *PHOX2A, CDH2, ADAMTS16, BTBD3,* and *IRS2* were hypomethylated in MM. They need to be discussed in more detail owing to their well-established role in oncogenesis and cancer progression. For example, *ADAMTS16* has been shown to promote gastric cancer growth [48]. Moreover, Sakamoto and colleagues conducted a study in which they demonstrated its oncogenic character in oesophageal cancer [49]. The overexpression of *PHOX2A* has been demonstrated in neuroblastoma, both in tumour samples and in neuroblastoma cell lines [50, 51]. Aberrant overexpression of *CDH2* has been proven to be directly involved in MM promotion. Vandyke and colleagues confirmed that *CDH2* was significantly upregulated in newly diagnosed MM [52]. Notably, therapeutic interference with *CDH2* inhibited MM growth in a mouse Model [53]. Methylome analysis identified candidate genes whose methylation profiles significantly differed between MGUS and MM. Although changing methylation levels is a very well-established regulatory mechanism, it is not the ultimate way to mediate gene expression. Therefore, gene expression at the mRNA or proteomic level, despite alterations in the methylome, such as promoter hypermethylation, may be different than expected. After considering these phenomena, to confirm our findings and investigate whether the expression (at the mRNA level) of the abovementioned genes actually differs between MM and MGUS, we performed qRT‒PCR analysis. The results revealed that the *PHOX2A* and *CDH2* genes were overexpressed in MM compared with MGUS. On the other hand, the tumour suppressor *HTATIP2* was downregulated in MM compared with its premalignant predecessor. Notably, according to a study by Dong and colleagues, *HTATIP2* downregulation results from promoter methylation and predicts poor clinical outcomes in gliomas. Moreover, they demonstrated that the methylation inhibitor 5-aza-2'deoxycytidine restored *HTATIP2* expression in vivo [54]. Targeting epigenetic alterations as a target for novel anti-MM agents are currently bei 498 investigated in clinical trials. First epigenetic drug registered for MM treatment is panobinostat, 499 pan-deacetylase inhibitor, which was shown to be an option for RRMM in combination with 500 BTZ and dexamethasone [55, 56]. Nonetheless, Purinostat mesylate, a highly selective HDAC I/II binhibitor, 28 502 was demonstrated to outperform the pan-HDAC inhibitor panobinostat in MM [57]. Moreover, in-vitro results as well as first in human preliminary 504 reports from ongoing 1a phase clinical trials revealed that another novel HDAC inhibitor, 505 bisthianostat, is well tolerated supporting further phase 1b studies [58]. Although HDAC inhibitors and other epigenetic agents are not in the mainstream of 507 MM research, they highlight the importance and potential clinical application of epigenetic 508 studies in MM. Analysis of miRNAs in patient plasma revealed a new approach to the pathogenesis of MM. Specifically, increased expression of miRNA208b-3p, miR-582-3p, miR-10b-3p, and miR-371b-5p was observed for the first time in patients with MGUS, with concomitant decreases in patients with MM. Moreover, these miRNAs are good biomarkers (AUC > 0.7). In various cancers, the above miRNAs have significant effects on their pathogenesis. For example, decreased expression of miR-582-3p and miR-582-5p strongly and positively correlated with advanced clinicopathological features and shorter bone metastasis-free survival in prostate cancer patients. Increased expression of miR-582-3p and miR-582-5p inhibited the ability of prostate cancer cells to invade and migrate in vitro and inhibited bone metastasis in vivo [59]. In patients with triple-negative breast cancer (TNBC), miR-371b-5p expression was reduced, and overexpression of miR-371b-5p significantly attenuated the growth, migration, and invasion of TNBC cells [60].

miR208b-3p is correlated with the pathogenesis of oncological diseases such as hepatocellular carcinoma and osteosarcoma. However, its action significantly differs across individual types of cancer. In the aforementioned hepatocellular carcinoma model, strong expression of miR208b was observed, which resulted in the inhibition of ARID2 gene expression. In vitro studies revealed that a reduction in miR-208-3p expression inhibited the proliferation and invasion of hepatocellular carcinoma cells [61]. The opposite results were obtained during the study of osteosarcoma. It has been shown that overexpression of miR-208b can reduce the proliferation of human osteosarcoma cell lines (U-2OS and Saos-2) by arresting cell cycle progression [62]. We observed a similar correlation in our study. miRNA208b-3p was highly expressed in patients with MGUS and expressed at low levels in patients with MM. This may suggest that, with development to the fully symptomatic stage, the expression of this miRNA decreases, which consequently increases the expression of target oncogenes such as *CDH2*. The data obtained may indicate that the gradual reduction in miR208b-3p may be an unfavourable prognostic factor and significantly contributes to the development of MM from the preclinical stage. However, in vivo studies on MGUS and MM models should be performed to confirm this hypothesis.

The findings mentioned above, i.e., the upregulation of *PHOX2A* and *CDH2* accompanied by a decrease in the expression of *HTATIP2* in MM compared with MGUS, are consistent with the trends observed in the methylation and miRNA analyses. However, it should be noted that these observations are based on correlations, and the direct causal effects of the identified epigenetic alterations on gene expression have not yet been experimentally confirmed at the protein level. Therefore, these genes are potentially involved presumably involved in the MGUS-MM transition further functional validation is required to establish definitive mechanistic links. There is an urgent need for further research in this area, especially since epigenetic changes, although powerful and complex, have been shown to be reversible [63–65]. The reversibility of epigenetic changes is of paramount importance, particularly in cancer research. This well-established paradigm delineates research areas that need to be explored in detail. In particular, the results obtained may open many doors for potential therapies that disrupt epigenetic changes and thus contribute to improving the clinical outcomes of patients. This approach was demonstrated to be effective in MM in vitro and in mouse models [66–68].

## Conclusions

The analysis focused on new data from the perspective of MM pathogenesis. Owing to the available knowledge, the presented approach is new and original, which significantly increases the scientific value of the presented analyses. This study revealed that the *PHOX2* and *CDH2* genes may play significant roles in the pathogenesis of MM. Compared with MGUS, MM patients presented increased expression, and the appropriate epigenetic modifications responsible for their regulation are presented. Importantly, the observed plasma miRNAs, such as miR-208b-3p and miR-587-5p, inhibit *CDH2* gene expression and may act as both biomarkers and new directions for implementing miRNAs in the treatment of fully symptomatic MM, as well as preventive measures to prevent its progression from MGUS.

### Study limitations

Although our study provides new information on the impact of methylation on the development of MM and provides a direction for further research, it is not without drawbacks. First, the relatively small sample size reduces the statistical power of the analyses and may affect the robustness of the results, including receiver operating characteristic (ROC) analyses, which are prone to instability in smaller cohorts. Second, although we confirmed altered expression of several genes at the RNA level, no data on protein expression or functional assays were available, which limits the ability to fully establish the biological relevance of these alterations. Third, the cross-sectional design and lack of long-term clinical follow-up restrict the assessment of temporal dynamics and potential prognostic implications of the observed changes. However, the obtained results allowed the selection of important biological processes, genes, and miRNAs whose expression is worth examining in a larger group of patients. The patients included in the study formed a heterogeneous group in terms of sex, comorbidities, and age, but this is a natural phenomenon for patients with MM and MGUS. This variability actually constitutes an added value for the conducted study because it allowed us to select miRNAs that change in most patients with disease progression, and their concentration is not dependent on the occurrence of other diseases or sex. These findings prove that the proposed biomarker candidates are more specific for MM progression.

## Data Availability

The datasets used and analyzed during the current study are available from the corresponding author upon reasonable request.
